# Beyond mortality: years of life lost as an underestimated outcome in traumatic brain injury

**DOI:** 10.3389/fnhum.2026.1898591

**Published:** 2026-09-24

**Authors:** Walter Gomes da Silva Filho, Rodrigo Miranda Groberio, Miller Lucas de Faria, Vitória Caroline Reinoso de Almeida, Layza Julhia do Nascimento Moura, Bárbara Vargens Broedel, Kamila Aguiar Kröhling, Jéssica Aparecida Ludwig, Jader Vinicius da Silva Rocha, Rayane de Souza Moura, Rodrigo Freire de Almeida, Fernando Zanela da Silva Arêas

**Affiliations:** 1 Neurorehabilitation and Neuromodulation Laboratory, Department of Physiological Sciences, Federal University of Espírito Santo, Vitória, ES, Brazil; 2 Postgraduate Program in Physiological Sciences, Federal University of Espírito Santo, Vitória, ES, Brazil; 3 Baylor Scott and White Research Institute, Baylor Scott and White Institute for Rehabilitation, Dallas, TX, United States

**Keywords:** epidemiology, global burden of disease, long-term outcomes, traumatic brain injury, years of life lost

## 1 Introduction

Traumatic brain injury (TBI) remains one of the leading causes of death and long-term neurological disability worldwide, disproportionately affecting adolescents and adults in their most productive years of life (Areas et al., 2019; Dewan et al., 2018). Advances in acute care have significantly improved short-term survival; however, millions of individuals live for decades with the consequences of TBI (Areas and Gonçalves, 2022). Despite this reality, the impact of TBI is still predominantly assessed using short-term outcomes, such as in-hospital mortality, early functional scales, and length of stay.

While these indicators are essential for acute management, they fail to capture the long-term burden of TBI across the life course. Survival after the acute phase does not necessarily translate into full recovery or preservation of life expectancy. A growing body of evidence indicates that individuals who survive TBI face an increased risk of late mortality, progressive functional decline, and heightened vulnerability to neurological, psychiatric, and systemic conditions (Fazel et al., 2014; Bertotti et al., 2023; Gonçalves et al., 2025). Nevertheless, these outcomes remain largely invisible in both clinical research and health metrics that guide decision-making.

In this context, years of life lost (YLL) emerge as a particularly relevant outcome to capture the longitudinal impact of TBI. By accounting for the age at death relative to a standardized life expectancy, YLL provides a more sensitive estimate of premature mortality than crude death counts alone (Murray and Lopez, 2013). Although YLL is a core component of the Global Burden of Disease (GBD) framework, it has not been systematically incorporated into TBI-focused research (GBD 2019 Diseases Injuries Collaborators, 2020). In this opinion article, we argue that YLL should be adopted as a central outcome in TBI research, clinical reasoning, and health policy, supported by evidence from both retrospective and prospective perspectives.

## 2 YLL in the global burden of disease framework

YLL were developed within the framework of the GBD studies as a standardized metric to quantify premature mortality attributable to specific diseases and injuries (Murray and Lopez, 2013; GBD 2019 Diseases Injuries Collaborators, 2020). The YLL metric captures not only the occurrence of death but also the timing of death in relation to an optimal life expectancy, thereby providing a more comprehensive assessment of mortality-related burden. Conceptually, YLL is calculated as the difference between the age at death and a reference life expectancy obtained from standardized life tables that represent ideal survival conditions rather than country-specific mortality patterns. In the most recent GBD iterations, this reference life expectancy corresponds to a life expectancy at birth of approximately 86 years, derived from the lowest observed age-specific mortality rates across populations worldwide. The use of this normative life table ensures comparability across countries, regions, and time periods, avoiding bias introduced by local variations in mortality profiles.

By assigning a higher number of lost years to deaths occurring at younger ages, YLL explicitly incorporates the disproportionate social, economic, and human impact of premature mortality. Unlike crude or age-specific mortality rates, which treat all deaths equivalently regardless of age, YLL enables meaningful comparisons across diseases and injuries with distinct epidemiological and age-distribution patterns. For this reason, YLL has become a cornerstone indicator in global health research and a central component of burden-of-disease analyses conducted by the GBD consortium and the World Health Organization (Murray and Lopez, 2013).

This perspective is particularly critical in the context of TBI. Traumatic brain injury disproportionately affects children, adolescents, and young adults, resulting in a substantial number of deaths occurring far below the reference life expectancy (Areas et al., 2019; Dewan et al., 2018). Consequently, even relatively modest increases in TBI-related mortality can lead to disproportionately large losses of potential life years. Analyses restricted to death counts or short-term survival rates therefore systematically underestimate the true magnitude of TBI-related harm, whereas YLL-based assessments more accurately capture its long-term societal and population-level impact.

Mathematically, YLL were calculated by summing, across all ages, the product of the number of deaths at each age and the corresponding remaining life expectancy derived from the GBD standard life table. This approach is formally expressed as:


$$\begin{array}{l} YLL=\overset{n}{\underset{i=1}{\sum }}\left(N_{i}\times L_{i}\right) \end{array}$$

where *N*_*i*_ represents the number of deaths occurring at age or age group *i*, *L*_*i*_ denotes the remaining life expectancy at that age according to the GBD reference life table, and *n* corresponds to the total number of age categories analyzed. When individual-level data on age at death were available, YLL was equivalently computed as the sum of the differences between the GBD reference life expectancy at birth (approximately 86 years) and the age at death for each individual, ensuring consistency with the aggregated formulation and full comparability with GBD estimates.

## 3 Retrospective evidence and the hidden loss of life years after TBI

Large population-based retrospective studies consistently demonstrate that individuals who survive a traumatic brain injury experience a sustained increase in long-term mortality compared with age- and sex-matched individuals from the general population (Fazel et al., 2014; Bertotti et al., 2023; Harrison-Felix et al., 2004; Brown et al., 2004; Ventura et al., 2010). Importantly, this excess mortality persists for years or even decades after the initial injury, suggesting that TBI should be understood as a chronic health condition rather than a time-limited acute event.

The mechanisms underlying late mortality after TBI are multifactorial. Neurological sequelae such as epilepsy, cognitive impairment, and neurodegeneration interact with systemic consequences, including autonomic dysfunction, metabolic alterations, and increased cardiovascular risk (Gonçalves et al., 2025, 2023; Masel and DeWitt, 2010; Gardner and Yaffe, 2015). Psychiatric disorders, substance use, and social vulnerability further amplify mortality risk, particularly among younger individuals injured during adolescence or early adulthood (Fazel et al., 2014; Harrison-Felix et al., 2004).

Despite these findings, most clinical and epidemiological studies remain anchored to short follow-up horizons, frequently restricted to hospital discharge or 6-month functional outcomes. As a result, premature mortality occurring years after injury is rarely attributed to TBI in burden estimates, reinforcing the misconception that survival of the acute phase represents an adequate measure of success (Brown et al., 2004; Ventura et al., 2010).

Incorporating YLL into retrospective analyses provides a more integrative assessment of this hidden burden. By quantifying premature death relative to expected lifespan, YLL captures the cumulative downstream effects of neurological injury, comorbidity accumulation, and social disadvantage. This approach aligns TBI research with contemporary models of chronic disease burden and supports a re-evaluation of outcome hierarchies traditionally used in the field.

## 4 The value of prospective approaches for capturing YLL in TBI

Prospective cohort designs offer a critical methodological advantage for quantifying YLL, as they allow systematic long-term follow-up and reduce the risk of misclassification of late deaths (Sterne et al., 2016; Hernán and Robins, 2016). Unlike retrospective studies that rely on administrative coding or death registries alone, prospective approaches can integrate clinical, functional, and social determinants of health across the life course.

In the context of TBI, prospective measurement of YLL enables the investigation of how injury severity, secondary insults, access to rehabilitation, and post-acute care trajectories shape long-term survival (Gonçalves et al., 2025; Whiteneck et al., 2016; Corrigan et al., 2010; Zaloshnja et al., 2008). Emerging evidence suggests that disparities in follow-up care and social reintegration may contribute substantially to excess mortality, particularly in low- and middle-income settings (Dewan et al., 2018; Hyder et al., 2007).

Moreover, embedding YLL as a predefined outcome facilitates stratified analyses across clinically meaningful subgroups, such as mild vs. severe TBI, pediatric vs. adult injury, and isolated vs. polytrauma cases. This level of granularity is essential for identifying high-risk populations and for evaluating whether specific interventions meaningfully preserve life expectancy rather than merely delaying death.

From a methodological perspective, the inclusion of YLL in prospective TBI research aligns injury science with global burden-of-disease frameworks and strengthens the external validity and policy relevance of long-term outcome studies.

## 5 Clinical implications

Incorporating YLL as a clinical outcome expands the understanding of TBI beyond immediate survival. This perspective underscores the importance of long-term follow-up, secondary prevention, and integrated care models aimed at reducing late mortality. It also challenges clinicians to redefine therapeutic success, shifting from a discharge-centered view toward strategies that preserve life expectancy and long-term health.

Importantly, discharge from acute care or rehabilitation should not be interpreted as the end of care or as equivalent to complete recovery. TBI may result in persistent neurological, cognitive, behavioral, and functional consequences that require continued monitoring after patients return to the community (de Koning et al., 2015). Long-term follow-up should therefore be individualized according to injury severity, residual disability, comorbidities, and patient needs, with coordination across acute care, rehabilitation, primary care, and relevant multidisciplinary services. Such continuity may also facilitate the identification of late complications and deaths that would otherwise remain disconnected from the long-term trajectory following TBI. From this perspective, incorporating YLL into longitudinal outcome assessment may encourage healthcare systems to extend the concept of successful TBI care beyond hospital survival and discharge.

## 6 Implications for research

From a research standpoint, adopting YLL as a central outcome necessitates changes in study design, follow-up duration, and analytical strategies. Future investigations should systematically quantify premature mortality and compare YLL across interventions and populations. Such an approach enables more robust evaluations of the true impact of emerging therapies and care pathways in TBI.

## 7 Implications for public health policy

An additional challenge is the substantial heterogeneity in post-acute TBI care across countries and healthcare systems. Following acute hospitalization, patients may return directly home, undergo inpatient or outpatient rehabilitation, transition to long-term care, or receive community-based services (de Koning et al., 2015; Zarshenas et al., 2019). The relative use and availability of these pathways vary according to healthcare organization, rehabilitation capacity, resource availability, geographic location, socioeconomic conditions, and mechanisms of healthcare financing (Htwe et al., 2024; World Health Organization, 2024). Consequently, no single proportion of patients returning home or receiving post-acute rehabilitation can adequately represent TBI care globally. Moreover, discharge destination alone should not be considered a surrogate for recovery, as patients returning home may continue to experience neurological, cognitive, behavioral, or functional impairments requiring ongoing care (de Koning et al., 2015).

These differences are particularly relevant when interpreting YLL across populations. Healthcare systems with limited access to rehabilitation, fragmented post-discharge follow-up, or incomplete linkage between hospital and mortality records may face greater difficulties in characterizing long-term outcomes after TBI (Htwe et al., 2024; World Health Organization, 2024). Conversely, systematic longitudinal follow-up and integration between acute, rehabilitation, primary, and community care may improve the identification of late mortality and provide a more complete understanding of the long-term burden associated with TBI. Thus, incorporating YLL into public health surveillance should be accompanied by consideration of the healthcare context in which deaths and long-term outcomes are observed.

## 8 Barriers and limitations to the implementation of YLL in TBI

Despite its potential value, several factors may explain why YLL has not been routinely incorporated as an outcome in TBI clinical practice and research. YLL was primarily developed as a population-level measure of premature mortality, whereas TBI outcomes have traditionally focused on acute mortality, neurological status, functional recovery, and disability (Murray and Lopez, 2013; GBD 2019 Diseases Injuries Collaborators, 2020). Its broader implementation therefore requires reliable information on age at death, appropriate standardized life tables, accurate mortality ascertainment, and mechanisms for linking acute clinical information with long-term outcomes.

Longitudinal follow-up represents an additional challenge. Patients with TBI may transition through multiple settings after acute hospitalization, including inpatient rehabilitation, outpatient rehabilitation, long-term care, primary care, and home- or community-based services (de Koning et al., 2015). Fragmentation between these settings may hinder continuous outcome surveillance, while loss to follow-up can limit the identification and interpretation of late deaths. These challenges may be particularly relevant in healthcare systems with limited rehabilitation capacity, incomplete mortality registries, restricted access to specialized follow-up, or substantial geographic and socioeconomic inequalities (Htwe et al., 2024; World Health Organization, 2024).

Cross-country comparisons also require caution. Although standardized reference life tables facilitate comparisons of YLL between populations, differences in healthcare organization, access to rehabilitation services, and post-acute care pathways may influence long-term outcomes following TBI (Htwe et al., 2024; Zarshenas et al., 2019; World Health Organization, 2024). Therefore, YLL should be interpreted alongside information describing the healthcare context and the characteristics of the population being studied.

Finally, YLL quantifies premature mortality but does not directly capture the substantial burden of disability, cognitive impairment, functional dependence, or reduced quality of life experienced by many TBI survivors. For this reason, we propose that YLL should complement rather than replace established clinical and functional outcomes. Integrating mortality-based measures such as YLL with disability, functional, cognitive, and quality-of-life outcomes may provide a more comprehensive representation of the lifelong burden of TBI.

## 9 Final considerations

Traumatic brain injury has traditionally been evaluated through short-term outcomes that prioritize immediate survival (Areas et al., 2019; Areas and Gonçalves, 2022). Accumulating evidence indicates that this approach substantially underestimates the long-term burden of TBI (Fazel et al., 2014; Bertotti et al., 2023; Gonçalves et al., 2025). YLL represent a critical metric for capturing premature mortality and the true impact of TBI across the lifespan (Murray and Lopez, 2013; GBD 2019 Diseases Injuries Collaborators, 2020). Integrating YLL into research, clinical practice, and public health policy constitutes a necessary conceptual shift to align TBI care with the complexity of its long-term consequences.
